# Supplementary material for: KLF6 and STAT3 co-occupy regulatory DNA and functionally synergize to promote axon growth in CNS neurons
Source: Sci Rep. 2018 Aug 22;8:12565. doi: 10.1038/s41598-018-31101-5 (PMC6105645; doi:10.1038/s41598-018-31101-5)
Supplement: Supplementary file 1 — Supplementary information [file 41598_2018_31101_MOESM1_ESM.pdf]

KLF6 and STAT3 co-occupy regulatory DNA and functionally synergize to promote axon growth in CNS neurons

Zimei Wang , Vatsal Mehra , Matthew Simpson , Brian Maunze , Advaita Chakraborty, Lyndsey Holan , Erik Eastwood , Murray Blackmore, Ishwariya Venkatesh

Figure S1

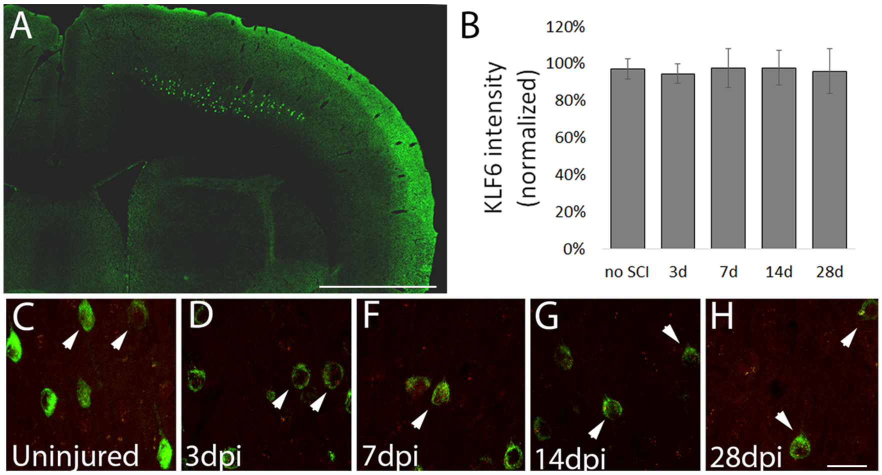

Figure S1. KLF6 expression in adult corticospinal tract neurons is insensitive to cervical axotomy. Mice ten weeks of age received C4/5 dorsal hemisections and injection of the retrograde tracer CTB-488. (A) CTB-488 label (green) in cortex identifies corticospinal tract projection neurons. (B-H) Immunohistochemistry for KLF6 (red) was performed, and the average intensity of KLF6 in corticospinal tract neurons (CTB-488, arrows) was normalized to the average intensity of cells in adjacent, uninjured layers of cortex. KLF6 intensity was dim prior to injury, and no change was detected at any post-injury time-point, indicating an unchanged level of KLF6 expression in injured corticospinal tract neurons ($P>.05$, ANOVA with Tukey, N=200 cells from 3 animals at each time point). Scale bars are 1mm (A) and 20μm (C-H).

Figure S2

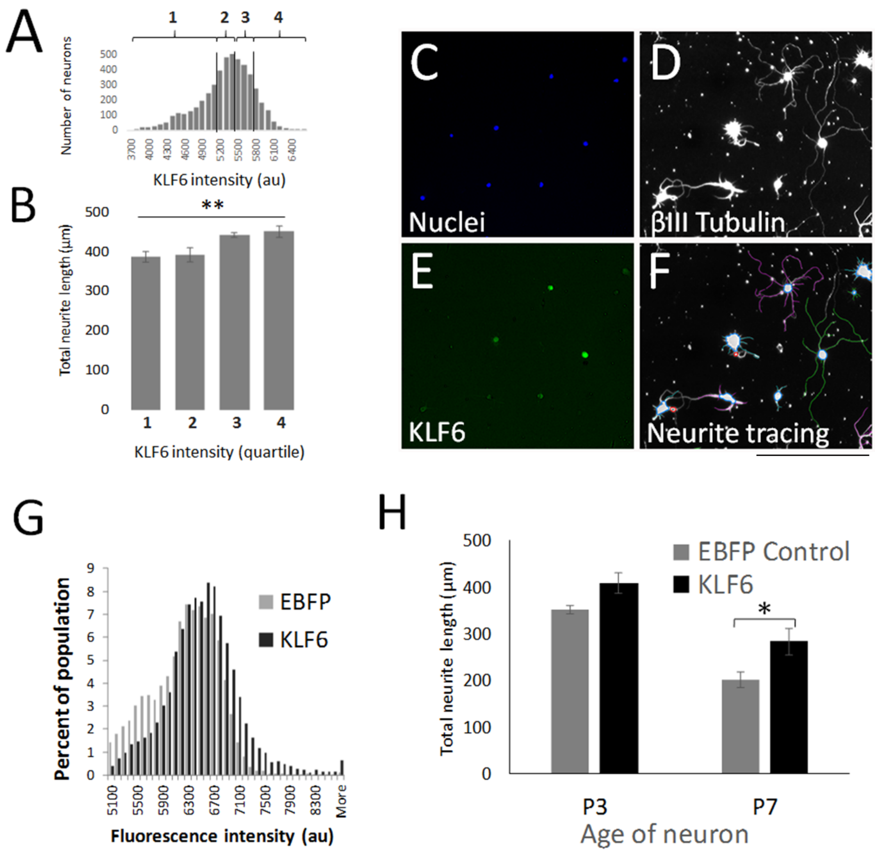

Figure S2. KLF6 expression correlates with and is sufficient to promote neurite outgrowth *in vitro*. (A,B) Cortical cells were prepared from P7 cortex, maintained in culture for two days, and immunohistochemistry for endogenous KLF6 (red) and neuron-specific βIII tubulin (white) performed. More than 10,000 cells were analyzed with a high content screening microscope. A naturally occurring distribution of KLF6 intensities was evident (histogram, A). Neurite outgrowth was averaged for the four quartiles of KLF6 expression. The top two quartiles were significantly longer than the bottom two ($p<.01$, ANOVA with TUKEY). (C-H). Postnatal cortical neurons were transfected with plasmid encoding EBFP control or KLF6. Overexpression of KLF6 (green) was confirmed by immunohistochemistry (arrows, E), and automated microscopy identified and traced neurites (F). Quantification of more than 10,000 individual neurons confirmed a population-level shift in KLF6 expression after transfection (histogram, G). Overexpression of KLF6 significantly increased average neurite lengths in P7 cortical neurons (*$p<.05$, ANOVA with Sidak's multiple comparisons, N>200 neurons in three separate experiments). Scale bar is 100µm.

Figure S3

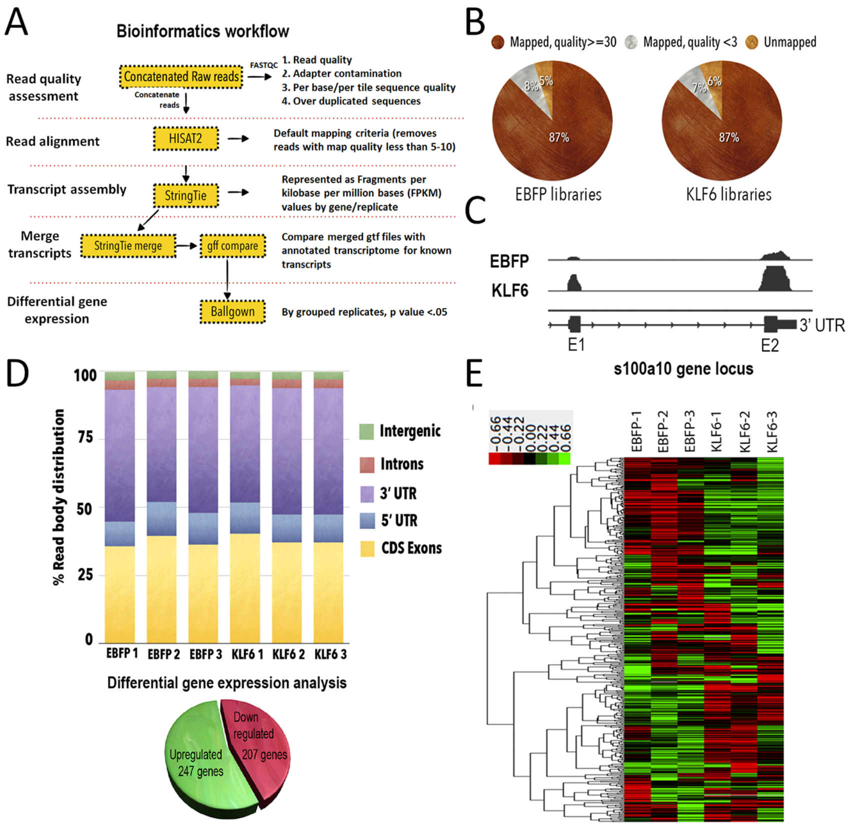

Figure S3. Transcriptional mechanisms underlying KLF6 mediated growth promotion investigated through RNA-Seq analysis. P5 cortical neurons were virally transduced to overexpress either EBFP control/ KLF6 and cultured on laminin substrates for 3 days before RNA extraction and deep sequencing. (A) RNA-Seq data analysis workflow (B) High mapping quality of sequenced reads confirmed library and sequencing quality (C) Representative IGV browser image of genomic locus corresponding to differentially expressed genes (s100a10). (D) Sequencing read distribution across the gene body for treatment groups were monitored to ensure optimal library and sequencing quality. Differential gene expression analysis identified 454 genes that significantly differ between control and KLF6 groups (p-value<0.05, FDR<0.05). 55% of the transcripts were upregulated and 45% of the transcripts were down-regulated in response to KLF6 over-expression. (E) Unsupervised hierarchical clustering showed gene expression signatures in control/ KLF6 groups and tight clustering among replicates.

Figure S4

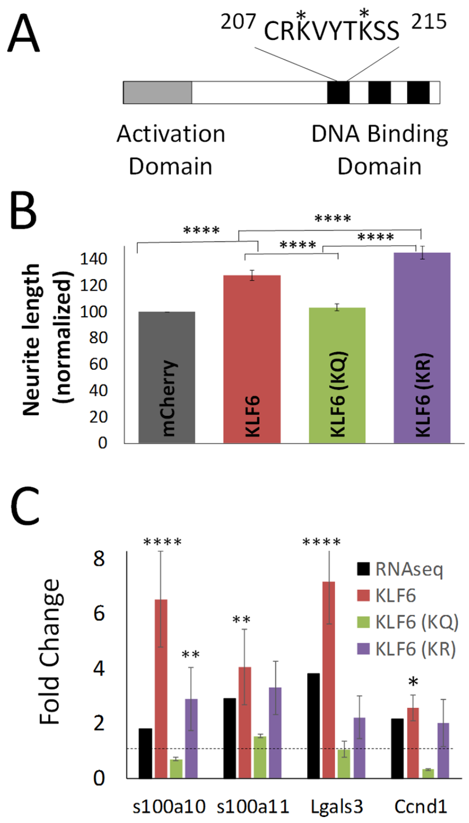

Figure S4. qPCR validation of transcriptional changes predicted by RNAseq. (A) Shows a schematic of KLF6 protein, indicating lysine residues shown previously to be subject to acetylation. Asterisks indicate lysines mutated to Q or R. (B,C) P7 cortical neurons were transfected with EGFP reporter and control mCherry, wildtype KLF6, or KLF6 with lysines 209 and 213 mutated to glutamine (Q) or arginine (R). After two days in culture, high content microscopy quantified neurite length in transfected neurons, and qPCR quantified expression of putative KLF6-regulated genes. (B) Wildtype KLF6 and KLF6(K,R), but not KLF6(K,Q) significantly increased neurite length (****p<0.0001, ANOVA with Tukey's multiple comparisons, N=>200 cells from three replicate experiments). (C) Wildtype KLF6 and KLF6(K,R) increased the expression of predicted KLF6-regulated genes, while KLF6(K,Q) did not (N=3, ****p<0.0001, **p<0.001, RM 2-way ANOVA with Sidak's multiple comparisons test).

Figure S5

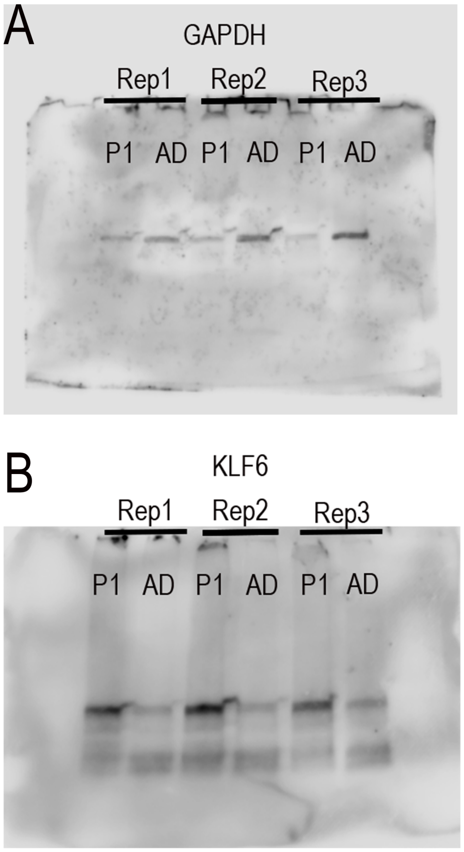

Figure S5. Western blotting for KLF6 in whole cortex revealed developmental decrease in protein expression (A) Full-length western blot showing bands corresponding to GAPDH loading control and KLF6 (B) in 3 biological replicates (whole cortex -P1 and Adult). Quantification of three replicate blots from three different sets of animals shows a significant reduction in KLF6 signal, normalized to GAPDH loading controls (**$p<.05$, ANOVA with Tukey, N=3).

Figure S6

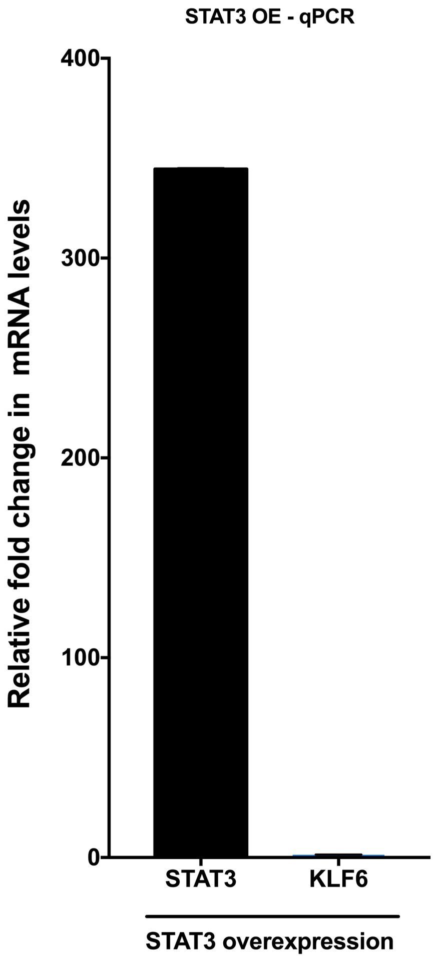

Fig S6. Quantitative PCR analysis revealed that KLF6 expression does not depend on STAT3 levels P5-6 cortical neurons were transfected to overexpress either EBFP control/ STAT3 and cultured on laminin substrates for 3 days before RNA extraction, cDNA synthesis and qPCR analysis. STAT3 plasmid overexpression resulted in ~300 level fold-change in STAT3 mRNA levels, but did not result in significant increases in KLF6 transcript levels. n=2 independent experiments, error bars = SEM.

TABLE legends

Sup_Table 1. List of differentially expressed genes
Sup_Table 2. Quantitative PCR primer sequences
